# The Glu27 genotypes of the Beta2-adrenergic receptor are predictors for severe coronary artery disease

**DOI:** 10.1186/1471-2350-7-31

**Published:** 2006-03-30

**Authors:** Khaled K Abu-Amero, Olayan M Al-Boudari, Gamal H Mohamed, Nduna Dzimiri

**Affiliations:** 1 Genetics Department, King Faisal Specialist Hospital and Research Centre (MBC – 03), P. O. Box 3354, Riyadh 11211, Saudi Arabia; 2 Department of Biostatics, Epidemiology and Scientific Computing, King Faisal Specialist Hospital and Research Centre (MBC – 03), P. O. Box 3354, Riyadh 11211, Saudi Arabia; 3 Biological and Medical Research Department, King Faisal Specialist Hospital and Research Centre (MBC – 03), P. O. Box 3354, Riyadh 11211, Saudi Arabia

## Abstract

**Background:**

The role of the Beta2-adrenoceptor (beta2-AR) Gln27Glu polymorphism in the manifestation of cardiovascular diseases is still unclear.

**Methods:**

In the present study, we evaluated the potential relevance of the c.79 C>G (p.Gln27Glu) polymorphism of this receptor gene for coronary artery disease (CAD) and its associated risk factors in Saudi Arabs. Genotyping was performed by PCR using the confronting two-pair primer (PCR-CTPP) method.

**Results:**

In the general population group (BD) (n = 895), 68.5% were homozygous wild-type C/C, 28.3% were heterozygous C/G and 3.2% were homozygous mutant G/G. Among the CAD patients (n = 773), 50.6% were homozygous wild-type C/C, 43.6% were heterozygous C/G and 5.8% were homozygous mutant G/G, while in the angiographed control group (CON) (n = 528), 71.8% were C/C, 24.4% C/G and 3.8% G/G genotypes. These results indicate that both the C/G (*p *= < .001) and G/G (*p *= .005) genotypes are significantly associated with CAD, when compared to the CON group. In addition, C/G (*p *= < .001) and G/G (*p *= < .001) were significantly associated with CAD, when compared to the BD group. Furthermore, stepwise logistic regression showed that the genotype [C/G (*p *< .001) and G/G (*p *< .001)] increase the risk of CAD.

**Conclusion:**

These results shows that the Gln27Glu genotypes (homo- or heterozygous) of the beta2-AR may be independent predictors of severe CAD.

## Background

The β_2_-adrenoceptor (β_2_-AR) plays an important role in the parasympathetic and sympathetic regulation of heart rate and contractility both in intact cardiovascular system and disease [1]. Apart from its cardiovascular function, the β_2_-AR also exerts vasodilatory and relaxing effects on different types of vascular smooth muscle. Hence, mutations in the gene coding for this protein are likely to have a great impact on cardiovascular reactivity and function. The gene is highly polymorphic in humans, the most common of which are the three polymorphisms Glu27, Gly16 and Thr164 that have been invariably associated with different cardiac risk factors. Besides, there is conflicting data in the literature, specifically with regard to the relevance of the Glu27 polymorphisms for the manifestation of different cardiovascular diseases and in different populations. For example, while some investigators found an association between this polymorphism and obesity in French population [2], others failed to establish such a link in various other populations [3-5]. Besides, the Glu27 polymorphism was associated with rheumatoid arthritis [6], coronary events in elderly patients [7], elevated leptin and triglycerides levels [8], but not with hypertension [9-12], blood pressure and heart size [13], obesity [5] and DM [5]. These partly conflicting reports point to the likelihood that the relevance of these gene variants as susceptibility factors for acquiring CAD or its risk factors may vary among different ethnical groups. In order to test this notion, in this study we were interested specifically in evaluating the potential relevance of the Glu27 variant for CAD manifestation and its risk factors among Arabs, using the Saudi population as a study model. Our results indicate a strong association between the C/G and G/G genotypes of the β_2_-AR gene and CAD in Arabs. Therefore, these genotypes (C/G or G/G) could be used as a predictor of severe CAD at least in our population.

## Methods

### Study population

Three groups of Saudi individuals were recruited for the present study. The patient group comprised 773 candidates (477 males and 296 females; mean age 53.8 ± 1.08 yr) of Saudi Arabian descent with angiographically documented CAD (CAD group). The inclusion criteria for severe CAD involved, among others, the presence of angiographically established narrowing of the coronary vessels by at least 70%. A second group of 528 individuals (298 males and 230 females, mean age 52.3 ± 1.42 yr) undergoing surgery for heart valvular diseases and those who reported with chest pain, but were established to have clear vessels by angiography, were recruited as controls (CON) group. A further group of 895 healthy Saudi individuals (519 males and 376 females, mean age 50.5 ± 3.6 yr) visiting the Blood Donor Clinic at King Faisal Specialist Hospital and Research Centre from January 2003 to November 2004, were recruited as representing the whole spectrum of the Saudi general population (BD group). This study was performed in compliance with the Helsinki declaration and in accordance with the regulations laid down by the Hospital Ethics Committee (approval RAC # 2010020) and all participants signed an informed consent.

Five ml of peripheral blood were collected in EDTA tubes from all participating individuals after obtaining their written consent. DNA was extracted using the PURGENE kit from Gentra Systems (Minneapolis, MN, USA), and stored in aliquots at -20°C until required. Serum cholesterol and triglyceride levels were measured as routine in the main Hospital Pathology Laboratory. Triglyceride levels < 1.8 mmol/L and total cholesterol levels < 5.2 mmol/L were considered normal. Diabetic patients either had a known history of diabetes mellitus or were diagnosed according to the American Diabetes Association criteria [14]. Diagnosis of myocardial infarction was based on the consensus specified by the European society of cardiology and the American college of cardiology [15]. Information about all risk factors was procured either through patient interviews or by referring to their medical records.

### Determination of the β_2_-AR genotypes

The genotyping for the c.79 C>G (p.Gln27Glu) polymorphism of β_2_-AR was determined by polymerase chain reaction (PCR) procedure using a modified confronting two-pair primer (PCR-CTPP) method described previously [16]. As a quality control, we sequenced and confirmed the genotype status of 288 samples representing the three different genotypes.

### Statistical analysis

Genotype frequencies among the various groups were compared by Chi-Square test. Multivariable logistic regression was used to study the effect of the β_2_-AR genotype on CAD status, incorporating other variables, such as coronary risk factors, into the model. A two-tailed *p *value < .05 was considered statistically significant. All analyses were performed using the SPSS v.10 (SPSS Inc., Chicago, IL, USA) statistical analysis software.

## Results

In the BD group (n = 895), 613 (68.5%) were homozygous wild-type C/C, 253 (28.3%) were heterozygous C/G and 29 (3.2%) were homozygous mutant G/G. Among the severe CAD patients (n = 773), 391 (50.6%) carried the C/C genotype, 337 (43.6%) were heterozygous C/G and 45 (5.8%) and were homozygous for the G/G genotype. Comparison of the CAD with the BD group, using the C/C wild-type genotype as the reference, both the C/G and G/G genotypes were associated with CAD (*p *= < .001), see Table 1. Furthermore, the frequency of the Glu27 allele (G allele) was significantly higher in the CAD group (*p *= < .001), Table 1.

**Table 1:** Genotypic and allelic distribution of the *Gln27Glu *polymorphism of β_2_-AR gene among angiographically confirmed coronary artery disease patients (CAD) and blood donors group (BD). CI, confidence interval

	**CAD (n = 773)**	**BD (n = 895)**	**Odds ratio**	**95% CI**	***P *value**
**Genotypes**					
C/C	391 (50.6%)	613 (68.5%)	-	reference	-
C/G	337 (43.6%)	253 (28.3%)	2.09	[1.69 – 2.58]	< .001
G/G	45 (5.8%)	29 (3.2%)	2.43	[1.46 – 4.06]	< .001
**Alleles**					
C	1119 (72.4%)	1479 (82.6%)	-	reference	-
G	427 (27.6%)	311 (17.4%)	1.81	[1.53 – 2.15]	< .001

Among the CON group (n = 528), 379 (71.8%) were C/C, 129 (24.4%) were heterozygous C/G and 20 (3.8%) were homozygous for the G/G genotype. Comparison of the CAD and CON groups, using the C/C wild-type genotype as the reference showed that both the C/G and G/G genotypes were associated with CAD (*p *values of < .001 and .005, respectively), see Table 2. Furthermore, the frequency of the Glu27 allele (G allele) was significantly higher in the CAD group (*p *= < .001), Table 2.

**Table 2:** Genotypic and allelic distribution of the *Gln27Glu *polymorphism of β_2_-AR gene among angiographically confirmed coronary artery disease patients (CAD) and angiographed controls (CON). CI, confidence interval

	**CAD (n = 773)**	**CON (n = 528)**	**Odds ratio**	**95% CI**	***P *value**
**Genotypes**					
C/C	391 (50.6%)	379 (71.8%)	-	reference	-
C/G	337 (43.6%)	129 (24.4%)	2.53	[1.97 – 3.24]	< .001
G/G	45 (5.8%)	20 (3.8%)	2.18	[1.26 – 3.76]	.005
**Alleles**					
C	1119 (72.4%)	887 (84.5%)	-	reference	-
G	427 (27.6%)	163 (15.5%)	2.08	[1.69 – 2.55]	< .001

Univariate analysis showed that β_2_-AR genotypes (C/G and G/G), age and diabetes mellitus were associated with CAD, whereas elevated cholesterol (*p *= .313), elevated triglycerides (*p *= .220), family history of CAD (*p *= .395), gender (*p *= .057), hypertension (*p *= .894) and myocardial infarction (*p *= .614) were not, see Table 3. Further analysis of the data based the number of vessels affected showed no association with Glu27 allele distribution among the CAD group (results not shown). The variables in Table 3, showing an association (*p *= < .05) were then put into a stepwise logistic regression in order to study the possible combined effect of the genotypes with other risk factors on CAD manifestation. The only variable retained in the model was the genotype (*p *= < .001), see Table 4, pointing to an association of the genotypes C/G (*p *= < .001) and G/G (*p *= < .001) with severe CAD manifestation.

**Table 3:** Determination of odds ratio for the CAD risk factors among CAD and CON groups

**Risk factor**	***Status***	**CAD**	**CON**	**Odds ratio**	**[95% C.I]**	***p *value**
Genotype	C/C	391	379	reference	-	-
	C/G	337	129	2.53	1.96 – 3.27	< .001
	G/G	45	20	2.18	1.23 – 3.90	.004
Age	< 40	128	113	reference	-	-
	≥ 40	645	415	1.37	1.03 – 1.84	.027
Elevated Chol	No	138	106	reference	-	
	Yes	635	422	1.16	0.86 – 1.55	.313
Elevated TG	No	208	138	reference	-	-
	Yes	565	390	1.19	0.91 – 1.56	.188
DM	No	375	306	reference	-	-
	Yes	398	222	1.46	1.16 – 1.84	< .001
FH	No	540	372	reference	-	-
	Yes	233	156	1.03	0.80 – 1.32	.817
Gender	F	296	230	reference	-	-
	M	477	298	1.24	0.99 – 1.57	.057
Hypertension	No	86	60	reference	-	-
	Yes	687	468	1.02	0.71 – 1.47	.894
MI	No	543	364	reference	-	-
	Yes	230	164	0.94	0.73 – 1.20	< .614

Chol, Cholesterol; DM, Diabetes Mellitus; FH, family history; MI, Myocardial infarction on admission; TG, Triglycerides. Elevated serum triglycerides (> 1.8 mmol/L); Elevated total cholesterol (> 5.2 mmol/L).

**Table 4:** Stepwise multiple logistic regression results

**Risk Factor**	**# of CAD cases**	**Status**	**Odds ratio**	**[95% C.I]**	***p *value**
Genotype	391	C/C	reference	-	-
	337	C/G	2.5	1.79 – 3.49	< .001
	45	G/G	3.18	1.66 – 6.05	< .001

It is noteworthy that since the consanguinity rate in the Saudi population is > 65% [17], it was difficult for us to test for Hardy-Weinberg equilibrium because random mating among this population is not satisfied.

## Discussion

For determining the genotype frequencies of the β_2_-AR gene we selected 895 Saudi blood donors representing the whole spectrum of the Saudi population. To our knowledge this is the largest group thus far involved in studying the frequency of the β_2_-AR (Glu27) genotypes. The present study shows that the homozygous C/C genotype of this gene is the most predominant, while the G/G is the least prevalent in the Saudi population. The frequency of the C/G genotype (28.3%) was higher than the frequencies of 15, 16, 10, 13, 26 and 19% observed among Ghanaians, Kenyans, Sudanese, Filipinos, Indians and Chinese populations respectively [18]. However, the rate was lower than 32, 50, 53 and 48% observed among African Americans, Scottish, Caucasian USA and Swedish populations respectively [18].

The frequency for the G/G genotype was 3.2%, which is almost similar to the rate observed among Ghanaians, Filipinos and Indian populations respectively. However, our rate was lower than the rates of 21, 15, 14 and 11% observed among Scottish, Caucasian USA, Swedish and Sudanese populations respectively [18].

The observed rate of 17% for the Glu27 allele (G allele) is similar to the 21% found among the African-Americans [19], but appears to be higher than the 7 and 8% in the Chinese and Japanese [12] and lower than the frequency of 34% in Caucasian Americans [19]. Put together, these observations point to differences in the frequencies of the C/G and G/G genotypes and the Glu27 allele of the β_2_-AR gene among the different populations.

Our results also indicate strong association of both the C/G and G/G genotypes with the manifestation of severe CAD in Arabs. However, further studies in larger populations are required to verify these results. Nonetheless, it is noteworthy that, while this mutation has been associated with various CAD risk factors such as obesity [2], rheumatoid arthritis [6], coronary events in elderly patients [7], elevated leptin and triglycerides levels [8] in different populations, currently there is hardly any data available in the literature implicating it in the manifestation of CAD. Besides, as described previously, the data implicating different β_2_-AR polymorphisms in these diseases has been inconsistent, with some studies pointing to an association and others showing no relationship of these polymorphisms with various cardiovascular disorders. Our observation together with studies by other investigators furnishes support to the notion that the β_2_-AR Glu27 polymorphism may be invariably important for the manifestation of CAD in different populations.

## Conclusion

In summary, our results indicate a strong association between the C/G and G/G genotypes and the Glu27 (G) allele with severe CAD in Arabs. Therefore, these genotypes (C/G or G/G) could be used as a predictor of severe CAD at least in this population.

## Abbreviations

β_2_-AR Beta-2-adrenoceptor

BD Blood donors

CAD Coronary artery disease

Chol Cholersterol

CI Confidence interval

CON Control group

DM Diabetes mellitus

FH Family history of CAD

MI Myocardial infarction

PCR Polymerase chain reaction

TG Triglycerdies

## Competing interests

The author(s) declare that they have no competing interests.

## Authors' contributions

KKA was in charge of design and analysis of data, OMA performed the technical aspects of the study, PCR and genotyping, GHM performed the statistical analysis and ND was responsible for recruiting patients and overall supervision of the study.

## Pre-publication history

The pre-publication history for this paper can be accessed here:


